# Celiac disease and celiac serology in children with Type 1 Diabetes: diagnostic timing, serologic patterns, and implications for long-term management

**DOI:** 10.48130/pedi-0026-0005

**Published:** 2026-07-23

**Authors:** Michael Schnapper, Anat Guz-Mark, Manar Matar, Moshe Phillip, Shlomit Shalitin, Dror S Shouval, Noa Tal, Orith Wasibourd-Zinman, Noam Zevit, Raanan Shamir

**Affiliations:** 1 Institute of Gastroenterology, Nutrition and Liver Diseases, Schneider Children's Medical Center of Israel, Petach Tikva 4920235, Israel; 2 Gray Faculty of Medical and Health Sciences, Tel-Aviv University, Tel-Aviv 6997801, Israel; 3 The Jesse Z. and Sara Lea Shafer Institute of Endocrinology and Diabetes, National Center for Childhood Diabetes Schneider Children's Medical Center of Israel, Petah Tikva 4920235, Israel

**Keywords:** Celiac disease, Type 1 diabetes, Tissue transglutaminase

## Abstract

The prevalence of celiac disease (CeD) in children with type 1 diabetes mellitus (T1DM) is significant, and celiac serology screening is routinely done in T1DM patients. The sequence of CeD and T1DM diagnosis and the natural history of transient celiac serology in T1DM is poorly defined. We aimed to investigate the temporal relationship between T1DM and CeD diagnoses, and the long-term natural history of celiac serology and CeD diagnosis in children with T1DM. A retrospective cohort study of 1,799 children with T1DM, treated at a single tertiary center (2009–2024), was evaluated for clinical, laboratory, and histologic data. Patients with positive anti-tissue-transglutaminase IgA antibodies (anti-TTG) were stratified based on CeD diagnosis or long-term serological behavior on a gluten-containing diet. Among 136 patients with T1DM and positive anti-TTG, 113 were diagnosed with CeD, 15 had persistent seropositivity (without confirmed CeD), and eight had a single borderline result (< ×3 upper limit of normal). CeD was diagnosed after T1DM in 81.4% of cases, and prior to TIDM in 18.6%. Patients diagnosed with CeD prior to T1DM exhibited more gastrointestinal symptoms, higher EMA positivity, and poorer glycemic control (*p* < 0.05). HbA1c values at follow-up were significantly higher in patients with CeD, compared to those with a single borderline anti-TTG, *p* < 0.01. In conclusion, most, but not all, patients with T1DM and elevated celiac serology, eventually develop biopsy-confirmed CeD. CeD can precede T1DM in a significant number of cases, emphasizing the need for continued monitoring for celiac serology in children diagnosed with T1DM and monitoring for developing T1DM in patients with CeD.

## Introduction

Celiac disease (CeD) is a chronic autoimmune condition characterized by small intestinal inflammation in genetically predisposed individuals, triggered by the ingestion of gluten, a storage protein found in commonly consumed grains including wheat, barley, and related species. The only current treatment is a strict lifelong gluten-free diet (GFD)^[1]^. CeD may present with various gastrointestinal and extraintestinal manifestations but may also present at screening in asymptomatic individuals^[1,2]^. Diagnosis relies on serological testing as the initial screening tool, usually followed by histological confirmation with the finding of characteristic changes in small intestinal biopsies unless fulfilling criteria for non-biopsy diagnoses. The primary screening test involves the detection of IgA anti-tissue transglutaminase antibodies (anti-TTG) and, in cases of IgA deficiency, IgG-based serology^[3−5]^.

An association between CeD and other autoimmune conditions, particularly type 1 diabetes mellitus (T1DM), is well established^[6,7]^. ESPGHAN guidelines (2020) recommend screening all children with T1DM for CeD, reflecting the increased risk in this population^[4]^. A recent review further supports this approach, recommending repeat screening for CeD-negative patients with T1DM at 2 and 5 years after the diagnosis of diabetes, due to the higher likelihood of CeD developing within the first 5 years^[8]^. Positive celiac serology may be identified in up to 16% of children and adolescents with T1DM^[9−11]^. A recent meta-analysis demonstrated that the prevalence of CeD in children and adolescents with T1DM is 4–9 times higher than in the general population^[12]^. Over a decade ago, we reported a prevalence of CeD of 5.5% in patients with T1DM at our institution, only 26% of whom were symptomatic^[13]^. Interestingly, studies exploring the relationship between CeD and T1DM evaluated the occurrence of CeD at or after the diagnosis of T1DM^[14,15]^ but ignored the possibility that CeD diagnosed prior to T1DM may be prevalent as well.

The impact of CeD in patients with T1DM has been extensively studied, although findings remain inconsistent. Some studies suggest that adherence to GFD improves glycemic control and growth parameters, while others report only minimal or no significant benefits^[16−18]^. Most children diagnosed with CeD during diabetes follow-up are asymptomatic. However, since elevated anti-TTG can be a transient phenomenon, especially in patients with autoimmune diseases, there is concern whether TTG-based screening may lead to overdiagnosis of CeD in cases where a biopsy is not performed. The phenomenon of transient celiac serology in children with T1DM was previously reported in a short-term study by Waisbourd-Zinman et al.^[13]^ and was confirmed in several other studies^[19−21]^. However, the long-term natural history of transient TTG positivity in children with T1DM remains unclear.

The present study aimed to evaluate the natural history of CeD serology in patients with T1DM over a long-term period and to establish the temporal relationship in the timings of the T1DM and CeD diagnoses.

## Materials and methods

### Patients and setting

We conducted a retrospective cohort study of pediatric patients (aged 0–18 years) diagnosed with type 1 diabetes mellitus (T1DM), treated at a single tertiary pediatric center between 2009 and 2024. The total number of 1,799 T1DM patients was determined based on our institutional database, which includes all pediatric patients followed at our endocrine clinic during this period. Patients were identified using ICD-9 diagnostic coding for T1DM. Electronic medical records (EMRs) were reviewed to identify patients screened for CeD serology. The study included all pediatric patients diagnosed with T1DM during this period who underwent at least one TTG test during follow-up; patients lacking serological data were excluded (*n* = 309). All patients with an elevated result of anti-TTG were included in the study.

### Data and variables

Demographic and clinical data included age and sex, family history of autoimmune diseases, age and timing of T1DM and CeD diagnosis, gastrointestinal symptoms, and adherence to GFD among patients diagnosed with CeD.

Laboratory parameters included baseline anti-TTG (TTG0) and follow-up TTG (TTG1) for those still observed under gluten-containing diets; endomysium antibodies (EMA) and hemoglobin A1c (HbA1c) levels at the time of T1DM diagnosis, as well as one and two years post-diagnosis. Additional data included the presence of autoimmune markers related to T1DM: anti-insulin, anti-islet cell, and anti-glutamic acid decarboxylase (GAD) antibodies, as well as markers of thyroid autoimmunity, hemoglobin levels, and liver function tests.

Until November 2018, Anti-TTG antibodies were measured using IgA ELISA (IMMCO Diagnostics, Buffalo, NY, USA); since November 2018, TTG was measured using the BioPlex® 2200 Celiac IgA and IgG Kits (Bio-Rad Laboratories, Hercules, CA, USA). Anti-EMA was measured using immunofluorescence throughout the study period. Anti-EMA was measured using the indirect immunofluorescence assay (IMMCO, DiaSorin S.p.A, New York, NY, USA).

Patients were classified into three groups:

• Group 1 (T1DM + CeD diagnosis): patients with a confirmed diagnosis of CeD (based on the relevant ESPGHAN guidelines for diagnosis of CeD that were in place at the time of diagnosis).

• Group 2 (T1DM + fluctuating CeD serology): patients with fluctuating anti-TTG levels above the upper limit of normal, but without progression to confirmed CeD. These patients were followed while continuing gluten-containing diets during the study period.

• Group 3 (borderline single positive CeD serology + T1DM): patients with a single low-positive anti-TTG result (< 3× ULN) that normalized on follow-up, without a diagnosis of CeD, also on a gluten-containing diet.

Lastly, we followed up on a cohort of 17 patients published over a decade ago who had positive celiac serology when diagnosed with T1DM and who had negative celiac serology at short-term follow-up^[13]^.

### Data analysis

Statistical analyses were performed using IBM SPSS Statistics v29 (IBM Corp., Armonk, NY, USA). Categorical variables were compared between groups using chi-square or Fisher's exact tests. Continuous variables were compared between groups using the Kruskal–Wallis test. All statistical tests were two-sided, and a *p-*value < 0.05 was considered statistically significant.

## Results

### Cohort characteristics and group classification

The medical records of 1,799 patients under 18 years of age with T1DM, all treated at the Institute for Gastroenterology, Nutrition and Liver Disease and the Institute for Endocrinology at Schneider Children's Medical Center of Israel, were reviewed. All patients had anti-TTG testing performed during their follow-up. Of these, 136 patients had positive anti-TTG results and were included in the study.

Group 1 (T1DM + CeD) included 113 patients (59.3% female), with a mean age at T1DM diagnosis of 8.75 ± 4.23 years and at CeD diagnosis of 9.27 ± 4.57 years. Among this group, 92 (81.4%) patients were diagnosed with CeD after T1DM diagnosis, with a mean of +1.86 years (IQR: +0.4 to +2.8 years) between diagnoses. The remaining 21 (18.6%) patients were diagnosed with CeD prior to T1DM, with a mean interval of −5.00 years (IQR: −7.51 to –1.48 years) between diagnoses. The mean anti-TTG level at CeD diagnosis was 9.38× ULN (IQR: 1.1–14.0), with 76.4% of patients also showing EMA positivity as well (*n* = 14; 12.3% were EMA negative). Gastrointestinal symptoms were reported by 34.7%. A duodenal biopsy was obtained in 101 (89.8%) patients. In the remaining cases, biopsies were not performed due to parental objection, and although ESPGHAN guidelines exempt T1DM from its recommendations for a non-biopsy approach, the diagnosis was made according to the ESPGHAN non-biopsy criteria (4). After CeD diagnosis, 67.1% of patients reported good adherence to GFD, while 20.3% had poor adherence, and 12.6% lacked data to assess adherence.

Group 2 (T1DM + fluctuating CeD Serology) included 15 patients (65.2% female), in whom T1DM was diagnosed at a mean age of 7.36 ± 4.1 years. All patients had TTG > × 3 ULN at baseline and throughout follow-up, with a mean baseline TTG level of 4.3× ULN (IQR: 1.0–5.3), with 66.6% EMA positivity. Duodenal biopsies were obtained in six (40%) of patients in this group, all with normal histology.

Group 3 (T1DM + a borderline single positive CeD serology) consisted of eight patients (34.8% female), with a mean T1DM diagnosis age of 6.61 ± 3.03 years. The mean baseline TTG level in this group was 1.83× ULN (IQR: 1.0–2.7), with 25% EMA positivity. In all cases, repeated anti-TTG measurements were normal after the first borderline-positive measurement. Duodenal biopsies were normal in the single patient from whom they were obtained.

### Autoimmune conditions and family history

Autoimmune thyroid conditions were present in 7.4% of the whole cohort. A first-degree family member had a diagnosis of CeD in 13.2%. Similarly, 18.7% of patients had a first-degree relative with type 1 diabetes mellitus.

Several significant differences were found between patients with positive CeD who were diagnosed with CeD (Group 1) and patients with only transient positive serology from Groups 2 and 3, as detailed in Table 1. Baseline and follow-up TTG, EMA, and histology were all significantly higher in Group 1 compared to Groups 2 and 3 (*p* < 0.01). Gastrointestinal symptoms were present only in children diagnosed with CeD. HbA1c values at T1DM diagnosis, 1 year, and 2 years post T1DM diagnosis significantly differed between groups, with higher values in those with co-diagnosis of T1DM and CeD (*p* < 0.01 at all time points).

**Table 1:** Group comparisons.

Comparison	Group 1 (CeD + DM1) *n *= 113	Group 2 (transient serology and DM1) *n *= 15	Group 3 (one-time positive serology and DM1) *n *= 8	*p*-value
TTG0 mean (baseline) (× ULN)	9.38 (SD ± 14.84)	4.3 (SD ± 5.1)	1.83 (SD ± 0.9)	*p* < 0.01
EMA positive, *n* (%)	86, (76.4%)	10, (66.6%)	2, (25.0%)	*p* < 0.01
Biopsy performed, *n* (%)	101, (89.8%)	6, (40.0%)	1, (12.5%)	*p* < 0.01
TTG1 mean (follow-up while on GCD)	9.00 (SD ± 9.62)	4.9 (SD ± 5.09)	3.8 (SD ± 0.9)	*p* < 0.05
HbA1c at diagnosis (%)	11.34 (SD ± 2.35)	10.8 (SD ± 2.28)	10.6 (SD ± 2.95)	*p* <0.01
HbA1c at 1 year (%)	7.58(SD ± 1.00)	7.52 (SD ± 1.22)	7.49 (SD ± 1.20)	*p* <0.01
HbA1c at 2 years (%)	7.73(SD ± 1.01)	7.64 (SD ± 1. 7)	7.61 (SD ± 0.5)	*p* <0.01
Gastrointestinal symptoms, *n* (%)	32, (28.3%)	0, (0%)	0, (0%)	*p* < 0.01
Female, *n* (%)	67, (59.3%)	9, (60.0%)	3, (37.5%)	*p* = 0.729
Autoimmune markers, *n* (%)	108, (95.3%)	15, (100%)	7, (87.5%)	*p* = 0.42
Family Hx of CeD, *n* (%)	11, (9.7%)	1, (6.7%)	0, (0.0%)	*p* = 0.43
Family Hx of T1DM, *n* (%)	19, (16.8%)	0, (0.0%)	1, (12.5%)	*p* = 0.23
CeD, celiac disease; DM1, type 1 diabetes mellitus; TTG0, tissue transglutaminase antibody at baseline; SD, standard deviation; EMA, endomysial antibodies; TTG1, tissue transglutaminase antibody at follow-up; HbA1c, glycated hemoglobin; M/F, male/female; Hx, history; GCD, gluten-containing diet.				

No significant differences were found in sex distribution, autoimmune markers, thyroid autoimmunity, family history of CeD, family history of T1DM, hemoglobin levels, or liver function tests, as detailed in Table 1.

### Temporal pattern of diagnosis in patients with T1DM and CeD

Among patients in Group 1, several differences were observed between patients diagnosed with CeD before or after T1DM diagnosis (Fig. 1).

**Figure 1:**
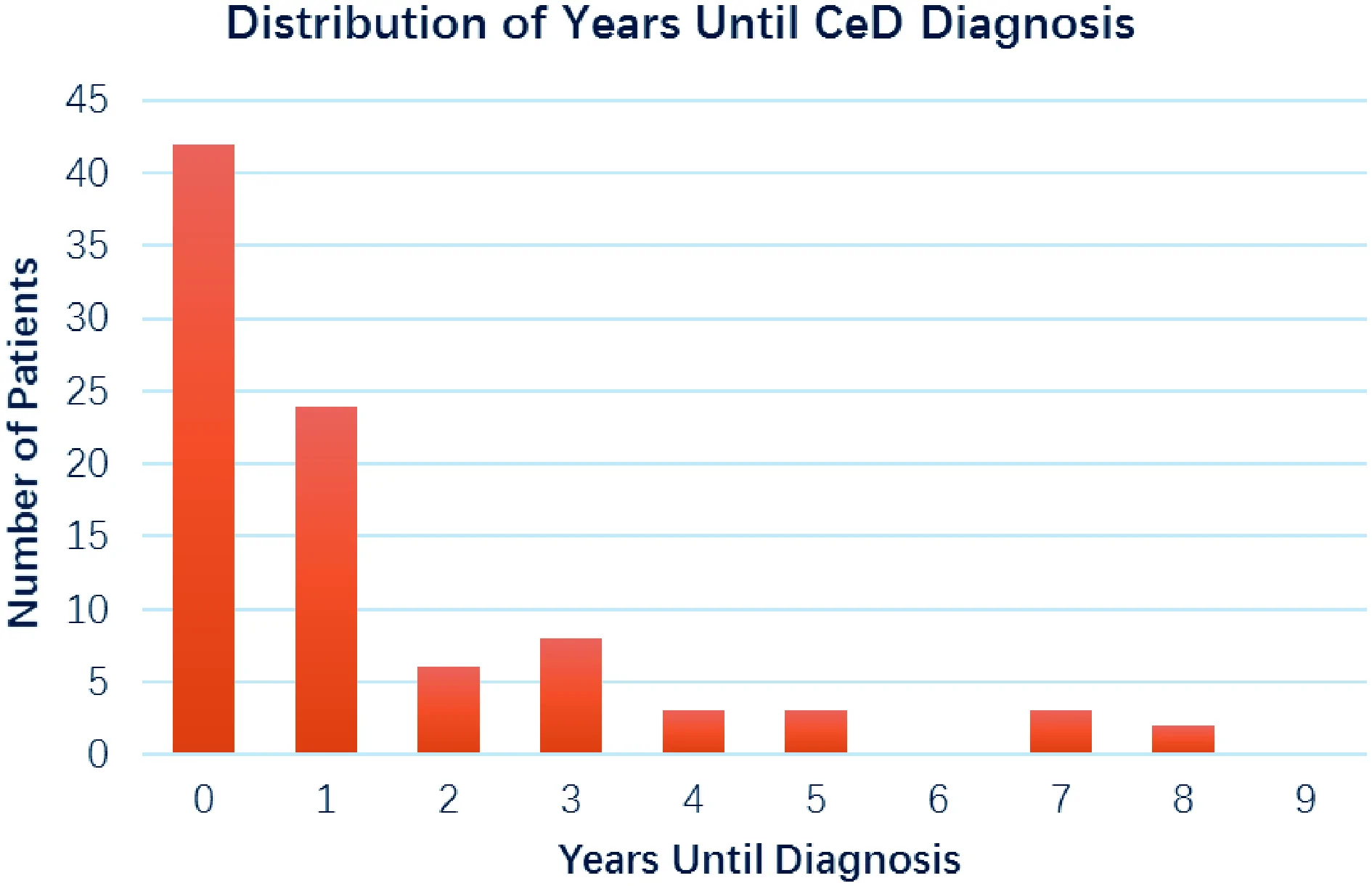
Distribution years from T1DM diagnosis to CeD diagnosis.

At the time of CeD diagnosis, patients who were diagnosed before T1DM had higher prevalences of gastrointestinal symptoms, higher rates of EMA positivity, and higher insulin autoantibody positivity (at the time of T1DM diagnosis) compared to patients diagnosed after T1DM, as detailed in Table 2. In contrast to insulin antibodies, thyroglobulin antibody positivity was more common in the CeD-before-T1DM group (Table 2). Significant differences were also seen in HbA1c levels at 1 year and 2 years post-T1DM diagnosis between the two groups (Table 2). Other variables, including family history of autoimmunity, liver enzymes, and microvascular complications (nephropathy, retinopathy), did not differ significantly by diagnosis order.

**Table 2:** Clinical and laboratory differences between patients diagnosed with CeD before vs after T1DM.

Comparison	CeD before T1DM	CeD after T1DM	*p*-value
GI symptoms at CeD diagnosis, *n* (%)	14/20 (70.0%)	18/91 (19.8%)	**0.001**
EMA positivity, *n* (%)	5/10 (50.0%)	5/54 (9.3%)	**0.001**
Thyroglobulin antibody positivity, *n* (%)	2/19 (10.5%)	0/87 (0.0%)	**0.002**
Insulin autoantibody positivity, *n* (%)	4/19 (21.1%)	40/87 (46.0%)	**0.046**
Liver function test abnormalities, *n* (%)	0/21 (0.0%)	2/92 (2.2%)	1.000 (NS)
Family history of CeD or T1DM, *n* (%)	8/21 (38.1%)	25/91 (27.5%)	0.33 (NS)
HbA1c at 1 year post-T1DM diagnosis, *n* (%)	Higher	Lower	**0.02**
HbA1c at 2 years post-T1DM diagnosis, *n* (%)	Higher	Lower	**0.008**
Microvascular complications (nephropathy, retinopathy)	None observed in either group	None observed in either group	N/A
Bold values are to indicate statistically significant *p*-values. CeD, celiac disease; T1DM, type 1 diabetes mellitus; GI, gastrointestinal; EMA, endomysial antibodies; HbA1c, glycated hemoglobin; NS, not significant; N/A, not applicable.			

### Follow-up on historical transient serology cohort

Of the 17 patients with a history of transient CeD serology in our previous study^[13]^, 11 (64.7%) showed spontaneous normalization of CeD antibodies without progression to CeD. Four patients (23%) were subsequently diagnosed with CeD, with an interval range of 8–17 years between T1DM diagnosis and CeD diagnosis. One patient was defined as having potential CeD (persistent positive serology, with normal duodenal biopsies), and one was lost to follow-up.

## Discussion

This study reports a large cohort of pediatric patients with co-diagnosis of T1DM and CeD. As described previously in the literature, in most cases, T1DM is diagnosed prior to CeD^[6]^; however, in this cohort we showed that a significant portion were diagnosed with CeD prior to T1DM. In addition, our findings reveal insights regarding the timing of CeD diagnosis in patients with T1DM, the risk of progression to CeD in cases of transient serologic responses, and the association between CeD and the risk of poor glycemic control as expressed by HbA1C levels.

### Timing of CeD in T1DM

A significant finding of our study is that approximately 19% of children with coexisting CeD and T1DM were diagnosed with CeD prior to the onset of diabetes. This deviates from the prevailing assumption in earlier literature that CeD typically develops after T1DM^[6]^. Even though it is more common for T1DM to precede the diagnosis of CeD, our findings highlight that a notable proportion may develop CeD beforehand, an observation also reported by Lindgren et al. in a recent Swedish nationwide longitudinal cohort study^[22]^, reporting CeD diagnosis prior to T1DM in 19%. Complementing this interplay between timing of diagnosis of CeD and T1DM, two decades ago, Ludvigsson et al. found that Swedish children with CeD had a 2.4-fold higher risk of developing T1DM before age 20, with elevated risks both in early childhood and later, although the overall risk of developing T1DM in children with CeD remained low^[23]^. In addition, this shift may reflect evolving diagnostic practices, greater awareness of CeD in young children, or changes in the natural history of autoimmune diseases in pediatric populations. Notably, patients diagnosed with CeD first presented more frequently with gastrointestinal symptoms and additional autoimmune markers, possibly suggesting a broader underlying immune dysregulation. The lower gastrointestinal symptomatology could be explained by the fact that most children were diagnosed within the first year from T1DM onset due to universal screening for CeD in children diagnosed with T1DM. Our observation may be due to the structure of our tertiary center allowing us to determine the time of CeD diagnosis in patients already diagnosed with T1DM who had negative celiac serology when diagnosed with T1DM. The finding that almost one of five children with CeD and T1DM developed T1DM after the diagnosis of CeD highlights the need for awareness among health care providers and families regarding the potential for T1DM onset in this population. Specifically, promoting symptom-based education and including fasting blood glucose levels as part of the laboratory evaluation in the follow-up of CeD.

### Possible effects of GFD on the development of T1DM

It has been hypothesized that GFD may have a protective role in the development of autoimmunity in patients with CeD; however, the evidence remains scarce. Our findings, consistent with the limited existing literature, suggest that the presence of CeD, even when diagnosed prior to T1DM and followed by adherence to a GFD, does not appear to prevent the subsequent development of T1DM. In a recent publication, Tiberti et al.^[24]^ reported that while a GFD in children with CeD was associated with a reduction in islet autoantibodies in some patients, others, particularly those with multiple islet autoantibodies at diagnosis, remained positive and eventually developed T1DM despite long-term adherence to the diet (20%). However, due to the lack of control groups in these studies, it is difficult to determine whether the observed changes were directly attributable to the GFD.

### Glycemic control and relationship to CeD diagnosis and time of diagnosis

Children diagnosed with both T1DM and CeD had higher HbA1c levels at both the time of diagnosis and at 1- and 2-year follow-ups, compared to children with only transient celiac serology but without a confirmed CeD diagnosis (Fig. 2).

**Figure 2:**
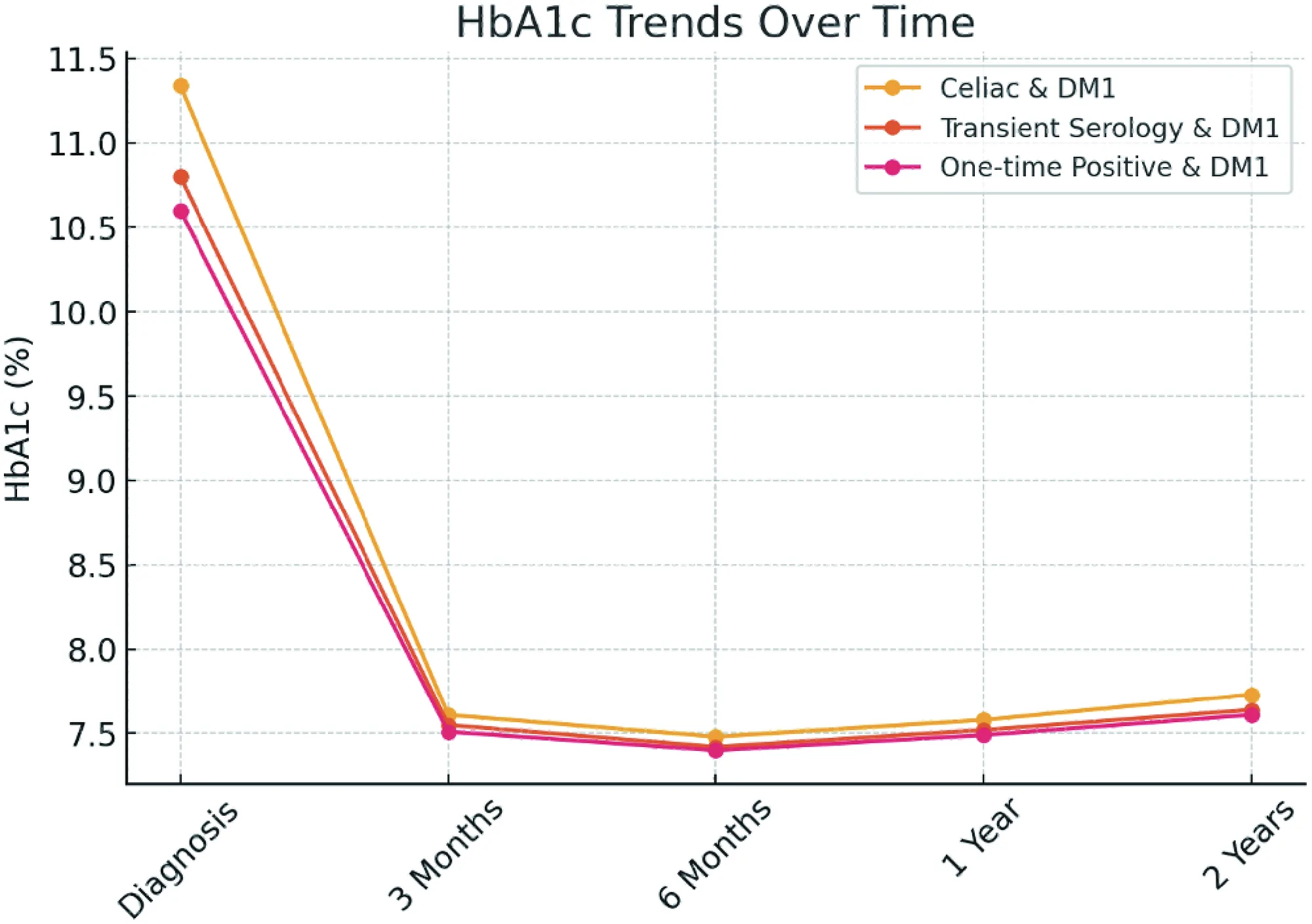
HbA1c trends over time in children with type 1 diabetes (DM1) according to celiac serology status.

Notably, those diagnosed with CeD prior to the onset of T1DM demonstrated even poorer glycemic control than those diagnosed with CeD after developing T1DM. This pattern may suggest that an earlier onset of CeD may contribute to metabolic dysregulation, possibly due to prolonged inflammation or nutritional deficiencies preceding diabetes onset. Another explanation would be that these differences may be due to a bias, as not all patients developed CeD in temporal proximity to T1DM diagnosis, which could contribute to better glycemic control in this subgroup. In addition, as GFD adherence in this cohort was suboptimal, reflected by only two-thirds of patients reporting good GFD adherence, this suboptimal glycemic control might reflect a pattern of incomplete nutritional adherence in children struggling with both diseases.

The effect of GFD on glycemic control in children with diabetes is controversial, with previous studies reporting mixed findings^[16−20]^. Several studies reported improved glycemic metrics after adopting GFD^[25−27]^. However, these studies often involved small sample sizes, limiting the generalizability of their findings. A recent systematic review reported no significant difference or negative effect on HbA1c by GFD in T1DM and CeD^[27]^. Conversely, a study by Sponzilli et al.^[28]^, which included controls, showed worsened HbA1c control in children with CeD and T1DM compared to controls with T1DM alone.

The observation in our cohort adds to the accumulating evidence on the glycemic control of patients with both diseases and its association with the sequence of diagnosis. It also serves to support the explanation to families as to why CeD diagnosis is important in children with T1DM.

### Transient serology and risk of progression to CeD

Our long-term follow-up revealed that the majority of patients with transient CeD serology eventually normalized their anti-TTG while remaining on gluten-containing diets. However, nearly a quarter do progress to biopsy-confirmed CeD over time. Notably, this outcome builds upon our earlier study and reflects a follow-up period of about 13 years, the longest time to our knowledge. This finding supports emerging evidence that transient celiac antibody elevations are not universally benign and highlights the importance of long-term monitoring for CeD serology in this population.

### Strengths and limitations

Our study strengths are the large cohort size, including children with T1DM who had negative celiac serology when diagnosed with T1DM, the comprehensive registry available at our center, and the availability of long-term longitudinal follow-up, enabling us to study the outcomes of transient celiac serology over more than a decade. Nevertheless, it has several limitations that should be acknowledged. First, its retrospective design carries an inherent risk of selection and information bias. Moreover, as the study was conducted at a single tertiary center, the findings may not be generalizable to broader populations. In addition, adherence to a gluten-free diet (GFD) was either self-reported or estimated by clinicians, which may introduce reporting bias. Finally, our analysis focused exclusively on patients with type 1 diabetes who had positive celiac serology, without including a comparison group of diabetic patients without celiac markers, limiting our ability to assess differences between these subgroups.

## Conclusions

This study emphasizes the need for vigilant, long-term monitoring of pediatric patients with T1DM and positive CeD serology, even if initially transient. Our cohort demonstrates that although most of these patients will not develop CeD, a notable subset will. Our findings also suggest that CeD precedes T1DM in a significant proportion of cases. This suggests that physicians taking care of children with CeD should be aware of this possibility, and better education of health care providers and families alike is needed for early identification of T1DM in children with CeD. Furthermore, metabolic outcomes appear to be influenced by the timing of CeD diagnosis, with earlier onset associated with poorer glycemic control. Achieving good medical and nutritional adherence for both diagnoses is both crucial and challenging in this population. Prospective studies are needed to further study the immunologic interplay between CeD and T1DM and to define optimal follow-up and treatment strategies.

## Data Availability

The datasets generated during and/or analyzed during the current study are not publicly available due to patient privacy and institutional restrictions but are available from the corresponding author on reasonable request.
